# Achieving Pain Control in Rheumatoid Arthritis with Baricitinib or Adalimumab Plus Methotrexate: Results from the RA-BEAM Trial

**DOI:** 10.3390/jcm8060831

**Published:** 2019-06-12

**Authors:** Peter C. Taylor, Yvonne C. Lee, Roy Fleischmann, Tsutomu Takeuchi, Elizabeth L. Perkins, Bruno Fautrel, Baojin Zhu, Amanda K. Quebe, Carol L. Gaich, Xiang Zhang, Christina L. Dickson, Douglas E. Schlichting, Himanshu Patel, Frederick Durand, Paul Emery

**Affiliations:** 1 Botnar Research Centre, University of Oxford, Oxford OX3 7LD, UK; 2 Division of Rheumatology, Northwestern University Feinberg School of Medicine, Chicago, IL 60611, USA; yvonne.lee@northwestern.edu; 3 Metroplex Clinical Research Center, University of Texas Southwestern Medical Center, Dallas, TX 75231, USA; RFleischmann@arthdocs.com; 4 Division of Rheumatology, Department of Internal Medicine, Keio University, Tokyo 162-5882, Japan; tsutake@z5.keio.jp; 5 Rheumatology Care Center, Birmingham, AL 35244, USA; eperkins@rheumatologycarecenter.com; 6 AP-HP, Pitie-Salpetriere Hospital, Dept of Rheumatology, Sorbonne Université, Pierre Louis Institute for Epidemiology and Public Health, 705013 Paris, France; bruno.fautrel@aphp.fr; 7 Eli Lilly and Company, Indianapolis, IN 46285, USA; zhu_baojin@lilly.com (B.Z.); amanda.quebe@lilly.com (A.K.Q.); gaich_carol_lynn@lilly.com (C.L.G.); zhang_xiang@lilly.com (X.Z.); dickson_christina_l@lilly.com (C.L.D.); schlichting_douglas_e@lilly.com (D.E.S.); himanshu.patel@lilly.com (H.P.); durand_frederick@lilly.com (F.D.); 8 Leeds Institute of Rheumatic and Musculoskeletal Medicine University of Leeds, Leeds LS1 3EX, UK; p.emery@leeds.ac.uk

**Keywords:** baricitinib, disease-modifying antirheumatic drugs, pain perception, outcomes research, patient perspective, rheumatoid arthritis

## Abstract

The purpose of the study was to assess the proportion of patients who achieve pain relief thresholds, the time needed to reach the thresholds, and the relationship between pain and inflammation among patients with rheumatoid arthritis (RA) and an inadequate response to methotrexate in RA-BEAM (NCT0170358). A randomized, double-blind trial was conducted, comparing baricitinib (*N* = 487), adalimumab (*N* = 330), and placebo (*N* = 488) plus methotrexate. Pain was evaluated by patient’s assessment on a 0–100 mm visual analog scale (VAS). The following were assessed through a 24-week placebo-controlled period: the proportion of patients who achieved ≥30%, ≥50%, and ≥70% pain relief, the time to achieve these pain relief thresholds, remaining pain (VAS ≤ 10 mm, ≤20 mm, or ≤40 mm), and the relationship between inflammation markers and pain relief. Baricitinib-treated patients were more likely (*p* < 0.05) to achieve ≥30%, ≥50%, and ≥70% pain relief than placebo- and adalimumab-treated patients, as early as Week 1 vs. placebo and at Week 4 vs. adalimumab. A greater proportion of baricitinib-treated patients achieved ≤20 mm or ≤40 mm remaining pain vs. placebo- and adalimumab-treated patients. Baricitinib-treated patients tended to demonstrate consistent pain relief independent of levels of inflammation control. In RA patients with an inadequate response to methotrexate, baricitinib provided greater and more rapid pain relief than adalimumab and placebo. Analyses suggest the relationship between inflammation and pain may be different for baricitinib and adalimumab treatments.

## 1. Introduction

Rapid, sustained pain control is a foremost goal for many patients with rheumatoid arthritis (RA); notably, in a survey approximately two-thirds of patients responded that pain was their treatment priority [1]. Many patients with RA who have achieved control of inflammation associated with good clinical response with RA therapy continue to report pain, including at levels described as moderate to severe [1,2,3,4]. This remaining pain may be a result of the multifactorial nature of pain associated with RA, which is not solely a result of inflammation; rather it may also be associated with structural damage, peripheral sensitization, or central amplification [2,5,6]. For other patients, despite a treat-to-target approach, the desired goal of remission cannot be attained. For these patients, ongoing pain is often a predominant symptom.

In RA-BEAM, a Phase 3 clinical trial of baricitinib, an oral, selective inhibitor of Janus kinase (JAK)1 and JAK2, baricitinib plus methotrexate (MTX) was associated with significant clinical improvements compared to patients treated with adalimumab plus MTX or placebo plus MTX. Baricitinib- and adalimumab-treated patients demonstrated similar improvement in swollen joint count (SJC), with both groups demonstrating significantly greater improvement relative to the placebo group beginning at Week 1 that was maintained through the placebo-controlled period (Week 24). For patient-reported pain, however, baricitinib-treated patients reported significantly greater improvements as early as Week 1 compared to placebo-treated patients, and as early as Week 2 when compared with adalimumab-treated patients. These statistical differences in pain relief between the active treatment arms were maintained through the duration of RA-BEAM (Week 52) [7]. This observation prompted us to explore differences in pain relief with baricitinib- and adalimumab-treated patients in RA-BEAM.

Publications of clinical trials in RA traditionally evaluate pain improvement only as central tendencies (i.e., mean change from baseline). To our knowledge, no prior reports have more fully characterized treatment effects or explored the relationship between the control of pain and inflammation with treatment. The objectives of this analysis were two-fold: first, to use the RA-BEAM data to assess the proportion of patients who achieve pain relief thresholds and the time needed to achieve these thresholds, and second, to investigate the relationship between inflammation and patient-reported pain.

## 2. Patients and Methods

### 2.1. Trial Design

The design and procedure of RA-BEAM have been described previously [7,8]. Briefly, RA-BEAM was a randomized, double-blind, double-dummy, placebo-controlled and active-controlled, parallel-arm, 52-week study conducted at 281 centers in 26 countries between 2012 and 2015 (ClinicalTrials.gov: NCT01710358). A total of 1305 patients on stable background MTX were randomly allocated (3:2:3) to placebo, adalimumab 40 mg, or baricitinib 4 mg. At Week 16, those patients considered non-responders received open-label rescue treatment with baricitinib 4 mg. After Week 16, patients may have received rescue treatment at investigator discretion. At Week 24, placebo-treated patients were switched to baricitinib. The study was conducted in accordance with the ethical principles of the Declaration of Helsinki and Good Clinical Practice guidelines. The study protocol was approved by each center’s institutional review board or ethics committee. All patients provided written informed consent.

### 2.2. Patients

Patients were ≥18 years old with active RA (≥6/68 tender and ≥6/66 swollen joints; serum high-sensitivity C-reactive protein (CRP) ≥6 mg/L). Patients had an inadequate response to MTX and either ≥3 joint erosions (based on radiographs), or ≥1 joint erosion with seropositivity for rheumatoid factor or anti-citrullinated peptide antibodies [7].

### 2.3. Pain Measures

Pain was measured with the patient’s assessment of pain visual analog scale (VAS), consisting of one question, “How much pain are you currently having because of your rheumatoid arthritis?” Responses range from 0 mm (no pain) to 100 mm (worst possible pain). The pain VAS was administered at every study visit.

#### Pain Thresholds

Our initial observation of differential pain response between baricitinib and adalimumab was based on mean change from baseline. We wanted to understand if these differences persisted when pain relief was evaluated against various thresholds of success, as is typical for other patient-reported outcomes in RA. Because there are no established, standard pain thresholds in RA, we reviewed the literature and selected two approaches. First, we applied percent change from baseline threshold recommendations from the general chronic pain literature, specifically those from the Initiative on Methods, Measurement, and Pain Assessment in Clinical Trials (IMMPACT), a multidisciplinary organization with the mission to develop consensus reviews and recommendations and to improve clinical trials of treatments for pain [9,10]. A 30% improvement threshold is described as “much improved, meaningful differences” and 50% represents “very much improved, substantial improvement” in chronic pain conditions. A 70% improvement threshold, although not defined in IMMPACT, was also evaluated because it is analogous to American College of Rheumatology response endpoints and was observed with patients in RA-BEAM. Second, while relative improvement is important, so is absolute pain; thus we evaluated thresholds of remaining pain (i.e., the absolute value of patient-reported pain) of ≤10 mm, ≤20 mm, or ≤40 mm, at Week 24. The ≤10 mm threshold reflects limited pain to no pain and is extrapolated from data by Wells et al. [11]. The ≤20 mm threshold represents a threshold when satisfaction with health is not negatively affected by pain [11,12]. The ≤40 mm threshold was derived from observed cut-off points between the pain VAS and the Patient Acceptable Symptom State (PASS) [13].

### 2.4. Outcomes

The proportion of patients achieving ≥30%, ≥50%, or ≥70% improvement from baseline by Week 24 was assessed, as was the median time when 50% of patients achieved these thresholds of pain relief. The proportion of patients achieving remaining pain VAS values of ≤10 mm, ≤20 mm, or ≤40 mm was assessed at Week 24. To evaluate if the differences in pain response were associated with inflammation, we assessed the relationship between levels of inflammation and pain relief at Week 24.

### 2.5. Statistical Analyses

All analyses were conducted with an intention-to-treat approach in which data from patients who received ≥1 dose of study drug were assessed, regardless of whether they completed the trial. Missing values were imputed with modified last observation carried forward for all analyses where applicable. Analyses were not adjusted for multiplicity.

Comparisons were made on the percent change in pain VAS from baseline to Week 24 using analysis of covariance (ANCOVA) and on the proportion of patients achieving pain relief at Week 24 between treatment arms using logistic models, adjusted for randomization factors (region, baseline joint erosion status (1–2 erosions plus seropositivity vs. ≥3 erosions)) and baseline pain VAS score. The median time needed for patients to achieve these pain relief thresholds were assessed through Week 24 using the cumulative incidence estimate with ‘competing risks’ which included rescue or discontinuation due to lack of efficacy before reaching the pain relief threshold. The Cox proportional hazards model with ‘competing risks’ (proportional sub-distribution hazards model) [14,15] was used to obtain the hazard ratio.

Remaining pain was analyzed across treatment groups with logistic regression models. Pain relief at Week 24 by CRP was evaluated using ANCOVA.

A mediation analysis with multiple mediators was conducted to evaluate the relationship between levels of inflammation and pain relief. The effects of change in inflammatory factors (CRP, erythrocyte sedimentation rate (ESR), SJC) as multiple mediators on change in pain outcome for each treatment over placebo during the 24-week period were evaluated in this analysis [16]. The total treatment effect on pain relief over placebo that can be accounted for by changes in CRP, ESR, and SJC in the mediation analysis is the ‘indirect’ or mediation effect, while the total treatment effect that cannot be accounted for by the ‘indirect’ effect is called the ‘direct’ effect. Observed data were used for the mediation analysis.

Statistical analyses were performed in SAS (SAS Institute; Cary, NC, USA, version 9.4). A two-sided *p* value < 0.05 was considered statistically significant.

## 3. Results

### 3.1. Pain Relief

As noted by Taylor et al. [7], patients had established and active RA. The mean baseline pain scores were well matched across treatment groups in this study and ranged from 60 to 62 mm with the median baseline pain of 62 mm [7]. Other baseline characteristics were well-balanced between the treatment arms [7]. A detailed description of the safety of baricitinib and adalimumab is available in the RA-BEAM publication [7]. In brief, adverse events were more frequent with baricitinib (71%) and adalimumab (68%) than with placebo (60%) through Week 24. Rates of serious adverse events through Week 24 were 5% with placebo, 5% with baricitinib, and 2% with adalimumab. 

As early as Week 1, significantly greater improvement in pain relief was observed between baricitinib and placebo (25% for baricitinib vs. 4% for placebo, *p* < 0.0001). At Week 24, the mean percentage reduction in pain from baseline for baricitinib, adalimumab, and placebo, respectively, were 51%, 39%, 17% (*p* = 0.001 for baricitinib and adalimumab vs. placebo and *p* = 0.030 for baricitinib vs. adalimumab).

**Figure 1:**
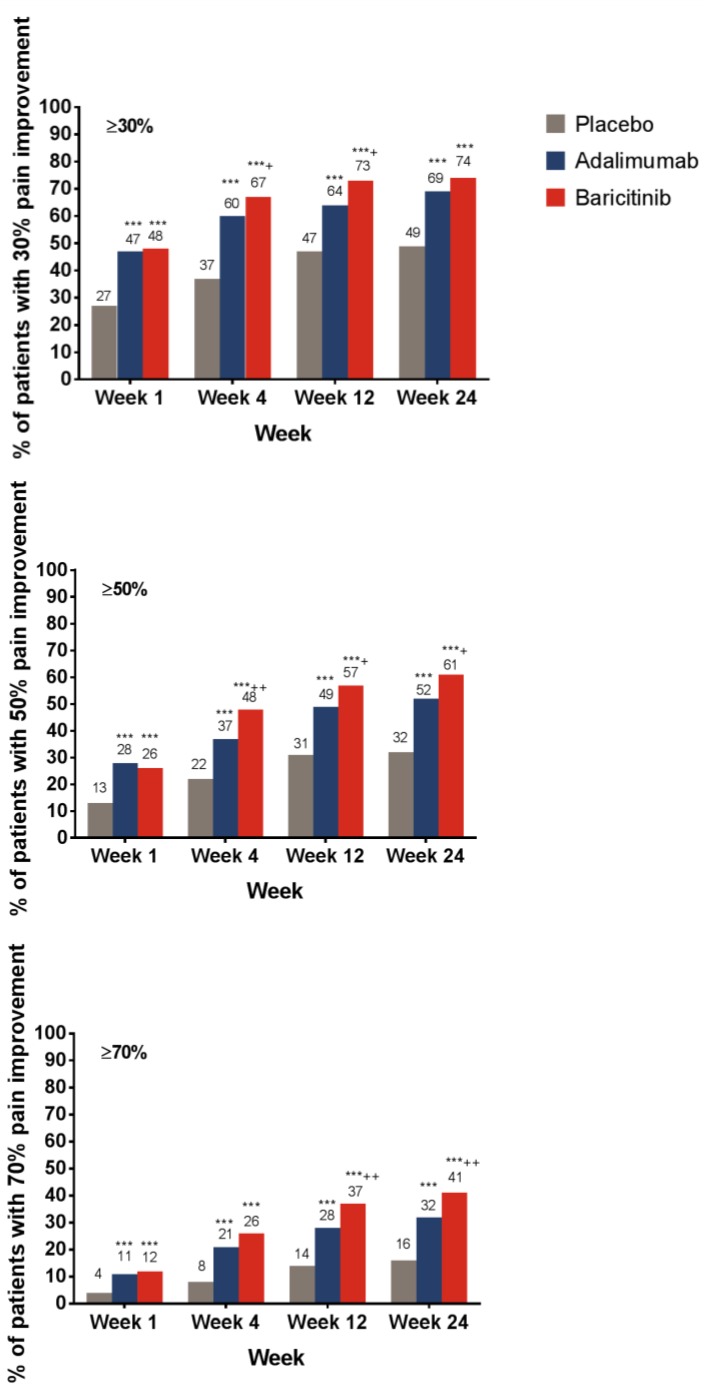
Percentage of patients who achieved pain relief thresholds from baseline, as measured by the pain VAS. *** *p* ≤ 0.001 vs. placebo; ^†^ *p* ≤ 0.05; ^††^ *p* ≤ 0.01, ^†††^ *p* ≤ 0.001 vs. adalimumab. Abbreviations: VAS = visual analog scale. Number of respondents who answered the pain question by week: placebo, *n* = 481 at Week 1 and *n* = 483 at all other weeks; adalimumab, *n* = 325 at Week 1 and *n* = 327 at all other weeks; baricitinib, *n* = 482 at all weeks.

A greater proportion of patients treated with baricitinib or adalimumab achieved the ≥30%, ≥50%, or ≥70% pain relief thresholds compared with placebo-treated patients at Week 1 (Figure 1). Compared with adalimumab-treated patients, a greater proportion (*p* < 0.05) of baricitinib-treated patients achieved ≥30% and ≥50% pain relief as early as Week 4 and ≥70% pain relief at Week 8. Differences between baricitinib and adalimumab for ≥50% and ≥70% pain relief were maintained through Week 24 (Figure 1). 

At Week 24, for the placebo-, adalimumab-, and baricitinib-treated patients, respectively, the proportion of patients who achieved ≥30% pain relief were 49%, 69%, and 74%; for ≥50% pain relief, the values were 32%, 52%, and 61%; and for ≥70% pain relief, the values were 16%, 32%, and 41%.

**Figure 2:**
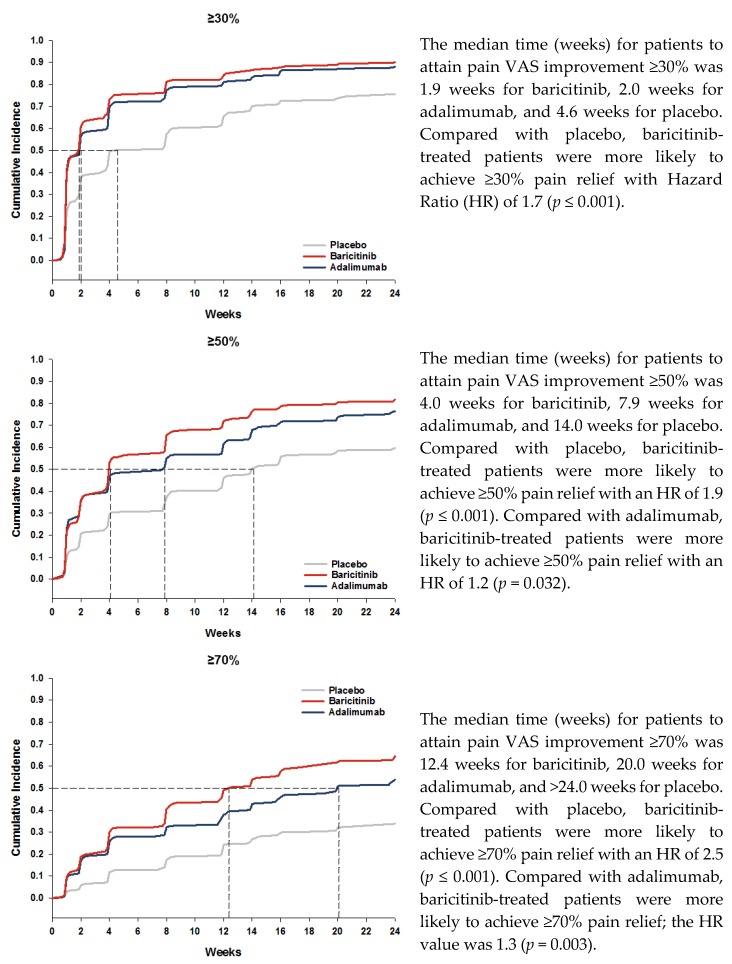
Time course for patients attaining pain relief thresholds.

The median time to achieve the ≥30% pain relief threshold was 2 weeks for baricitinib- and adalimumab-treated patients and 5 weeks for those on placebo (Figure 2). For ≥50% pain relief, the median time was 4 weeks for baricitinib, 8 weeks for adalimumab, and 14 weeks for placebo (Figure 2). For ≥70% pain relief, the median time was 12 weeks for baricitinib, 20 weeks for adalimumab, and >24 weeks for placebo (Figure 2). Compared with placebo, baricitinib-treated patients were more likely to achieve ≥30%, ≥50%, or ≥70% pain relief with Hazard Ratio (HR) values of 1.7, 1.9, and 2.5, respectively (*p* ≤0.001). Compared with adalimumab, baricitinib-treated patients were more likely to achieve ≥50% or ≥70% pain relief; the HR values for the ≥30%, ≥50%, or ≥70% pain relief thresholds, respectively, were 1.1 (*p* = 0.145), 1.2 (*p* = 0.032), and 1.3 (*p* = 0.003).

### 3.2. Remaining Pain

The differences in the proportion of patients who achieved the ≤40 mm, ≤20 mm, and ≤10 mm remaining pain thresholds were significantly greater for baricitinib and adalimumab compared with placebo as early as Week 1 (Table 1). Compared with adalimumab-treated patients, a greater proportion of baricitinib-treated patients achieved the ≤40 mm threshold (*p* ≤ 0.001) at Week 4, and a difference was observed between the active treatment groups at the ≤20 mm threshold by Week 12 (*p* ≤ 0.001; Table 1). Differences were maintained through Week 24. The percentage of patients who achieved the ≤10 mm remaining pain threshold was greater for baricitinib compared to adalimumab, but the difference reached statistical significance only at Week 12.

### 3.3. Relationship between Inflammation and Pain Relief

**Figure 3:**
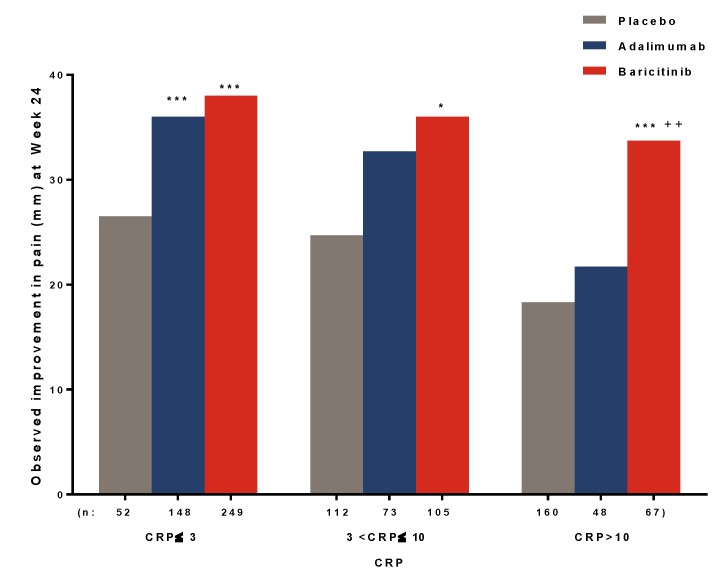
Pain improvement by remaining inflammation (CRP, mg/L) at Week 24. * *p* ≤ 0.05, *** *p* ≤ 0.001 vs. placebo; ^††^ *p* ≤ 0.01 vs. adalimumab. Abbreviations: CRP = C-reactive protein.

At Week 24, among patients with varying levels of inflammation, as measured by CRP as an objective marker of inflammation, baricitinib-treated patients tended to demonstrate consistent pain relief regardless of the CRP levels. In contrast, patients treated with adalimumab and placebo demonstrated less pain relief at higher CRP levels (Figure 3).

**Figure 4:**
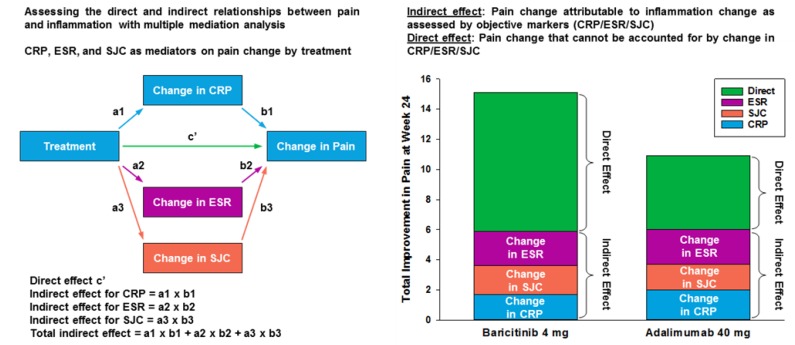
Relative contribution of inflammatory control to pain control.

While the total effect of baricitinib on pain relief over placebo at Week 24 was greater than that for adalimumab, changes in inflammation accounted for approximately 40% of the pain improvement with baricitinib and 50% of pain improvement with adalimumab (Figure 4; Table S1). In this analysis, the direct effects (i.e., those not associated with these markers of inflammation) of drug on pain relief were higher for baricitinib than for adalimumab after accounting for indirect inflammatory effects (Figure 4).

## 4. Discussion

In the RA-BEAM trial, patient-reported improvements in disease activity, physical function, and pain were greater for baricitinib plus MTX than for adalimumab plus MTX within 4 weeks of starting treatment and were maintained throughout the 52-week observation period [7]. In this analysis, we further explored pain relief experienced by patients. Baricitinib demonstrated greater and more rapid achievement of clinically significant levels of pain relief than adalimumab or placebo through Week 24. Furthermore, this differential effect became more marked as the pain relief thresholds increased, with approximately 40% of the patients receiving baricitinib achieving ≥70% pain relief from baseline by Week 24.

Another striking feature of this analysis was the rapid onset of effective mean pain relief at a cohort level with baricitinib plus MTX. Here, we show that for those patients achieving ≥50% or ≥70% pain relief, baricitinib had a shorter median time to achieving these pain relief thresholds than placebo or adalimumab. Specifically, for ≥50% pain relief, the 4 weeks needed for baricitinib was approximately half that of adalimumab treated patients (8 weeks). For patients achieving ≥30% pain relief, baricitinib and adalimumab had similar median time to onset (approximately 2 weeks). 

Remaining pain is commonly reported by patients with RA despite achieving satisfactory disease control by adopting the treat-to-target approach in disease management. A threshold of ≤20 mm remaining pain is considered to represent a point where health satisfaction is not adversely affected [11,12]. In our study, we found that patients treated with either baricitinib plus MTX or adalimumab plus MTX were significantly more likely to achieve ≤10 mm, ≤20 mm, and ≤40 mm thresholds for remaining pain compared to placebo plus MTX. Baricitinib separated from adalimumab by Week 4 for the ≤40 mm and by Week 8 for the ≤20 mm threshold. 

To explore the relative contribution of anti-inflammatory and other mechanisms of pain relief obtained with either baricitinib or adalimumab, we explored relationships between changes in patient-reported pain and an objective marker of inflammation, namely CRP. This analysis suggests that the difference in pain relief between baricitinib and adalimumab cannot be solely accounted for by differential effects on inflammation.

While it is clear from multiple clinical studies that baricitinib has a profound anti-inflammatory effect, as would be expected of a multi-cytokine inhibitor, these observations imply that JAK1 and JAK2 inhibition also has anti-nociceptive effects that are independent of at least certain aspects of the inflammatory process [17]. A rodent model indicated that treatment with baricitinib attenuates complete Freund’s adjuvant-induced joint deficits, a surrogate measure of joint pain [18]. At present, the mechanisms by which baricitinib modulates the pain experience independently of at least some generic features of inflammation is unknown. One possible mechanism could involve granulocyte-macrophage colony-stimulating factor (GM-CSF). GM-CSF is a cytokine that signals through JAK2 homodimers. In a rodent collagenase-induced instability model of osteoarthritis, pain was shown to be GM-CSF dependent, and therapeutic neutralization of GM-CSF rapidly and completely abolished arthritis pain [19]. Another possible pathway is through phosphorylation of signal transducer and activator of transcription 3 (STAT3). In rodent models of neuropathic pain following spinal nerve ligation, STAT3 phosphorylation was induced centrally in the dorsal spinal cord with upregulation of interleukin-6 (IL-6) mRNA in the dorsal root ganglia and elevated IL-6 concentrations in the dorsal spinal cord. Intrathecal administration of a JAK2 inhibitor blocked this STAT3 phosphorylation pathway with accompanying attenuation of both mechanical allodynia and thermal hyperalgesia [20]. It is known that the JAK-STAT3 system is activated through IL-6 signaling in spinal microglia and that this transduction pathway participates in development of pain associated with nerve alteration. However, it is not known whether such mechanisms have relevance to pain in established RA.

In rodent arthritis models, autoantibodies to citrullinated proteins (ACPA) are reported to induce joint pain independent of inflammation via a chemokine-dependent mechanism [21]. However, this is unlikely to account for the differences in pain relief between TNF blockade and JAK inhibition in our study as we did not observe statistically significant differences in ACPA change from baseline between baricitinib and adalimumab at Week 24.

This analysis has limitations. Specifically, the present findings represent post hoc analyses in which patients were not randomized according to their baseline pain. Pain studies may be complicated by the subjective experience of pain and the inherent limitations of a VAS as an instrument to measure pain experience. Additionally, the pain relief thresholds and remaining pain values are not yet firmly established for RA [12,13]. Further, some concomitant medication use (e.g., glucocorticoids) are controlled within a clinical trial, precluding assessment of any potential relationship between pain improvement and medication changes.

Pain relief with treatment may have clinical and holistic implications. Patients who experience pain relief are likely to report clinically significant improvements in other patient-reported outcomes, such as the Patient’s Global Assessment of Disease Activity and functional disability [22,23]. More broadly, reductions in pain have been associated with improvements in daily activity and work productivity [24]. In this analysis, we have presented evidence that baricitinib rapidly provides pain relief in patients with active RA on concomitant MTX to a magnitude greater than that observed with the TNF inhibitor, adalimumab. We have expanded prior research through a new and detailed analysis of the range of magnitude and kinetics of pain relief in a head-to-head study of baricitinib versus adalimumab and by exploring the relationship between the control of pain and inflammation with treatment. We observed that the inhibition of JAK1/JAK2 or TNF similarly ameliorate inflammatory markers, but the overall pain and the non-inflammatory component are faster and more markedly improved by baricitinib. Our findings merit further investigation into the biological mechanisms underlying pain relief.

The observations from this analysis may be of importance in managing the unmet needs of adequate pain relief in RA, whether in patients attaining the treatment targets of remission or low disease activity or in those who are unable to achieve these targets with biologic anti-TNF treatment. 

## Figures and Tables

**Table 1:** Percentage of patients who met the thresholds of remaining pain (VAS) over time by treatment groups. *** *p* ≤ 0.001 vs. placebo; ^†^ *p* ≤ 0.05; ^†††^ *p* ≤ 0.001 vs. adalimumab.

Threshold of Remaining Pain at Each Time Point (Week)	Placebo *n* (%)	Adalimumab *n* (%)	Baricitinib *n* (%)
**≤40 mm**			
1	150 (31)	132 (41) ***	208 (43) ***
4	193 (40)	169 (52) ***	298 (62) ***^,†††^
12	225 (46)	202 (62) ***	335 (69) ***^,†^
24	236 (49)	218 (66) ***	351 (73) ***^,†^
**≤20 mm**			
1	51 (11)	64 (20) ***	90 (19) ***
4	80 (17)	93 (28) ***	158 (33) ***
12	103 (21)	120 (37) ***	209 (43) ***^,†^
24	105 (22)	121 (37) ***	239 (49) ***^,†††^
**≤10 mm**			
1	20 (4)	32 (10) ***	40 (8) ***
4	29 (6)	49 (15) ***	88 (18) ***
12	52 (11)	63 (19) ***	124 (26) ***^,†^
24	56 (12)	86 (26) ***	144 (30) ***

## Supplementary Materials

The following are available online at https://www.mdpi.com/2077-0383/8/6/831/s1, Table S1: Multiple mediator analysis coefficients for adalimumab vs. placebo and baricitinib vs. placebo at Week 24.
