# Supplementary material for: Achieving Pain Control in Rheumatoid Arthritis with Baricitinib or Adalimumab Plus Methotrexate: Results from the RA-BEAM Trial
Source: J Clin Med. 2019 Jun 12;8(6):831. doi: 10.3390/jcm8060831 (PMC6617097; doi:10.3390/jcm8060831)
Supplement: Supplementary file 1 [file jcm-08-00831-s001.pdf]

Table 1. Multiple mediator analysis coefficients for adalimumab vs. placebo and baricitinib vs. placebo at Week 24.

**Multiple mediator analysis for adalimumab vs. placebo at Week 24**				
**Effect paths**	**Mediator**	**Coefficient**	**SE**	**p-value**
IV to Mediators (a paths)	CRP	-10.16	1.805	<.0001
IV to Mediators (a paths)	ESR	-12.20	1.960	<.0001
IV to Mediators (a paths)	SJC28	-1.996	0.455	<.0001
Direct Effects of Mediators on DV (b paths)	CRP	0.193	0.053	0.0003
Direct Effects of Mediators on DV (b paths)	ESR	0.190	0.049	0.0001
Direct Effects of Mediators on DV (b paths)	SJC28	0.840	0.184	<.0001
Total Effect of IV on DV (c paths)		-10.83	2.150	<.0001
Direct Effect of IV on DV (c' paths)		-4.881	2.124	0.0219
**Multiple mediator analysis for baricitinib vs. placebo at Week 24**				
**Effect paths**	**Mediator**	**Coefficient**	**SE**	**p-value**
IV to Mediators (a paths)	CRP	-14.29	1.626	<.0001
IV to Mediators (a paths)	ESR	-13.70	1.829	<.0001
IV to Mediators (a paths)	SJC28	-2.023	0.389	<.0001
Direct Effects of Mediators on DV (b paths)	CRP	0.122	0.048	0.0113
Direct Effects of Mediators on DV (b paths)	ESR	0.171	0.042	<.0001
Direct Effects of Mediators on DV (b paths)	SJC28	0.945	0.174	<.0001
Total Effect of IV on DV (c paths)		-15.15	1.917	<.0001
Direct Effect of IV on DV (c' paths)		-9.153	1.951	<.0001

IV: Independent Variable (treatment); DV: Dependent Variable (change in pain).
